# Performance of non-invasive fibrosis scores in non-alcoholic fatty liver disease with and without morbid obesity

**DOI:** 10.1038/s41366-021-00881-8

**Published:** 2021-06-24

**Authors:** Andreas Drolz, Stefan Wolter, Malte H. Wehmeyer, Felix Piecha, Thomas Horvatits, Julian Schulze zur Wiesch, Ansgar W. Lohse, Oliver Mann, Johannes Kluwe

**Affiliations:** 1 I. Department of Medicine, University Medical Center Hamburg-Eppendorf, Hamburg, Germany; 2 Department of General, Visceral and Thoracic Surgery, University Medical Center Hamburg-Eppendorf, Hamburg, Germany

**Keywords:** Epidemiology, Obesity, Body mass index

## Abstract

**Background:**

Non-invasive scores, such as the non-alcoholic fatty liver disease (NAFLD) Fibrosis Score (NFS), are increasingly used for liver fibrosis assessment in patients with NAFLD. The aim of this study was to assess the applicability and reliability of non-invasive fibrosis scores in NAFLD patients with and without morbid obesity.

**Methods:**

Three hundred sixty-eight patients with biopsy-proven NAFLD identified between January 2012 and December 2015 were studied; 225 with morbid obesity (biopsy obtained during bariatric surgery) and 143 patients without (termed as “conventional”).

**Results:**

Median age was 47 years, 57% were female. Median body mass index (BMI) was 42.9 kg/m^2^ with significant differences between our conventional and morbidly obese patients (BMI 29.0 vs. 50.8 kg/m^2^, *p* < 0.001). Overall, 42% displayed mild/moderate and 16% advanced liver fibrosis (stage III/IV). All tested scores were significantly linked to fibrosis stage (*p* < 0.001 for all). FIB-4 (AUROC 0.904), APRI (AUROC 0.848), and NFS (AUROC 0.750) were identified as potent predictors of advanced fibrosis, although NFS overestimated fibrosis stage in morbid obesity. Limiting BMI to a maximum of 40 kg/m^2^ improved NFS’ overall performance (AUROC 0.838). FIB-4 > 1.0 indicated high probability of advanced fibrosis (OR = 29.1). FIB-4 predicted advanced fibrosis independently from age, sex, BMI, and presence of morbid obesity.

**Conclusions:**

Our data suggest that FIB-4 score is an accurate predictor of advanced fibrosis in NAFLD throughout all BMI stages. Without adjustment, NFS tends to overestimate fibrosis in morbidly obese NAFLD patients. This problem may be solved by implementation of an upper BMI limit (for NFS) or adjustment of diagnostic thresholds.

## Introduction

As a consequence of the worldwide epidemic of obesity, diabetes mellitus, and metabolic syndrome, non-alcoholic fatty liver disease (NAFLD) has become one of the most frequent causes of chronic liver disease [1–4] with reported prevalence rates of up to 46% [5]. Without appropriate treatment, NAFLD and especially non-alcoholic steatohepatitis (NASH) can progress to fibrosis and ultimately cirrhosis. As a consequence of these developments, NASH has become the second leading etiology of liver disease in adults awaiting transplantation in the United States [6], and a major cause of hepatocellular carcinoma (HCC) [7].

Histological confirmation is considered the gold standard for diagnosis and staging of the disease [8, 9]. Stage of liver fibrosis is of paramount importance as it has been identified as an independent predictor of liver-related (and all-cause) mortality in patients with NAFLD in various studies [10–12]. Thus, detection of liver fibrosis is a crucial diagnostic step to stratify the individual risk of patients with NAFLD.

Several non-invasive tools have been introduced that allow assessment of fibrosis stage even without biopsy [9]. Transient elastography is among the most widely used techniques for non-invasive fibrosis assessment, but shows some limitations in morbidly obese patients [13, 14]. Fibrosis scores based on patient characteristics, anthropometric measurements, and laboratory parameters are increasingly used, and are considered as feasible alternative to imaging techniques, especially for exclusion of advanced fibrosis (stage III/IV) [9]. It has been repeatedly demonstrated that non-invasive fibrosis scores accurately predict advanced fibrosis in NAFLD [15–18]. To our knowledge, however, the value of non-invasive fibrosis scores with respect to body mass index (BMI) has not been evaluated.

The aim of this study was to assess the performance of different non-invasive scoring tools for liver fibrosis in NAFLD patients of different weight classes. For this purpose, we have evaluated non-invasive liver fibrosis scoring tools in a cohort of overweight or moderately obese (class I) NAFLD patients and in a NAFLD cohort with morbid or super (class III) obesity.

## Materials and methods

Patients with well-characterized and biopsy-confirmed NAFLD were retrospectively studied at the University Medical Center Hamburg-Eppendorf. All patients underwent biopsy between January 2012 and December 2015. Our patient population consisted of 143 NAFLD patients recruited by the Department of Gastroenterology (conventional cohort), and 225 patients with class III obesity who underwent bariatric surgery and were recruited by the Department of Surgery (morbidly obese cohort).

NAFLD was diagnosed in patients with hepatic steatosis on liver biopsy after exclusion of drug-induced steatosis, excessive alcohol consumption (>210 g/week in men or >140 g/week in women), chronic hepatitis B or C infection, and histological evidence of other concomitant chronic liver disease [15]. Alcohol abuse was excluded by interviewing the patients, and their relatives, if available.

Clinical and laboratory data were collected in the course of routine assessment prior to biopsy. Laboratory testing, including blood count, clinical chemistry, and coagulation-parameters, was performed at a median of 51 (interquartile range [IQR] 14–108) days prior to liver biopsy. If necessary, coagulation- and hematology tests were repeated shortly before the procedure. Within 48 h prior to biopsy, all patients underwent a physical examination including assessment of body weight and height; BMI was calculated according to the usual formula (BMI = body weight (kg) / height (m)^2^).

Obesity and overweight were defined by BMI ≥ 30 kg/m^2^ and BMI = 25–29.9 kg/m^2^, respectively [19]. Diabetes was diagnosed in patients already on anti-diabetic medication and in patients with a fasting glucose ≥126 mg/dl. Impaired fasting glucose (IFG) was defined by fasting glucose levels ≥110 mg/dl. Arterial hypertension was diagnosed in patients already under antihypertensive medication and in patients with a blood-pressure ≥130/≥85 mmHg.

This study was approved by the ethics committee of the Hamburg Medical Chamber (WF-042/17). The need for informed consent was waived due to the observational character of the study. A subgroup of our cohort has been previously evaluated with a different aim and methodology (PMID 29316577).

### Non-invasive fibrosis assessment

NAFLD Fibrosis Score (NFS) [15], aspartate aminotransferase (AST) to platelet ratio index (APRI) [20], AST/alanine aminotransferase (ALT) ratio, fibrosis 4 (FIB-4) score [21], and BARD score [17] were calculated based on clinical and biochemical parameters.

### Liver biopsy and histology

Liver biopsy was performed by mini-laparoscopy in 140 patients (38%), whereas 225 patients (61%) underwent intraoperative biopsy during bariatric surgery. Three patients (1%) underwent conventional percutaneous biopsy. Mini-laparoscopic biopsy was performed using an 18-gauge biopsy needle as described elsewhere [22]. Surgical liver biopsy (wedge biopsy) was performed during either laparoscopic Roux-Y gastric bypass or sleeve gastrectomy. Liver biopsy was performed when the liver appeared macroscopically abnormal at the time of bariatric surgery [23]. Pathological examination was performed at our central pathology department by experts in liver pathology. Liver fibrosis was evaluated according to the NASH-CRN scoring system: F 0 = no fibrosis; F I = perisinusoidal or portal/periportal fibrosis, F II = perisinusoidal and portal/periportal fibrosis, F III = bridging fibrosis, and F IV = cirrhosis [24]. Fibrosis stages 3 and 4 (F III/IV) were considered as advanced fibrosis. The NAS score was assessed based on the histological criteria of steatosis, ballooning, and inflammation, as described elsewhere [25].

### Statistical analyses

Data are presented as median (25–75% IQR) for metric variables or as absolute number (%). Continuous variables were compared using Mann–Whitney *U* test, binary variables via *χ*^2^ analysis or Fisher’s exact, as appropriate. Factors associated with fibrosis stage were evaluated by spearman rank correlation and by univariate ordinal regression. Area under receiver operating characteristic (AUROC) analyses were performed to identify cut-off values for different parameters with respect to the presence of advanced fibrosis. In addition, a precision-recall curve and ROC curve was used to visualize and assess predictive abilities of various parameters. Sensitivity, specificity, accuracy, positive and negative predictive values, as well as positive and negative likelihood ratios were calculated according to the usual formulae. Diagnostic odds ratio was calculated as described elsewhere [26]. AUROCs were compared by a non-parametric approach suggested by DeLong et al. [27]. Percent improvement in prediction error was calculated according to the following formula: $100 \times \frac{{\left[ {\rm{AUROC}}_{{\rm{Test}} - {\rm{Score}} - {\rm{AUROC}}_{\rm{Reference}} - {\rm{Score}}} \right]}}{{\left[ {1 - {\rm{AUROC}}_{\rm{Reference}} - {\rm{Score}}} \right]}}$, where Test-Score refers to the first (superior) and Reference-Score to the second (inferior) score. A *p* value < 0. 05 was generally considered statistically significant. SPSS Statistics Version 22 (SPSS Inc., Chicago, IL) and R (RStudio Version 1.2.1335) were used for statistical analyses.

## Results

### Patients

Three hundred sixty-eight patients with NAFLD were identified during the observation-period. Main clinical, histologic, and laboratory characteristics of our NAFLD cohort are illustrated in Table 1. Most of our patients were Caucasian (90%) with a median age of 47 years. A total of 43% of our patients were male. The majority of patients were overweight/obese (16%/77%) with typical underlying conditions suggestive of metabolic syndrome. We observed significant differences between the conventional and the morbidly obese cohort, attributable to different recruitment procedures. Accordingly, 88% (*n* = 126) of patients showed abnormal aminotransferase levels in the conventional group, compared to 32% (*n* = 73) in the morbidly obese group.

**Table 1:** Characteristics of 368 patients with non-alcoholic fatty liver disease.

Parameter	NAFLD patients (*n* = 368)			
	Overall	Conventional cohort (*n* = 143)	Morbidly obese cohort (*n* = 225)	*p* value
*Age, years (IQR)*	47 (35–56)	52 (35–60)	45 (35–51)	<0.001
*Sex (females), n (%)*	210 (57)	62 (43)	148 (66)	<0.001
*Concomitant diseases*				
Preexisting diseases				
Diabetes mellitus*, n* (%)	133 (36)	39 (27)	94 (42)	<0.01
Arterial hypertension*, n* (%)	226 (61)	68 (48)	158 (70)	<0.001
Hyperlipidemia*, n* (%)	182 (50)	28 (20)	154 (68)	<0.001
Findings during evaluation for biopsy				
Impaired fasting glucose or diabetes, *n* (%)	179 (49)	58 (41)	121 (54)	<0.05
Total cholesterol ≥200 mg/dl*, n* (%)	143 (39)	64 (45)	79 (35)	0.064
Triglycerides ≥170 mg/dl*, n* (%)	203 (55)	76 (53)	127 (56)	0.535
*Body mass*				
Height, m (IQR)	172 (165–179)	174 (164–181)	172 (165–178)	0.182
Weight, kg (IQR)	126 (94–158)	87 (75–100)	147 (128–170)	<0.001
BMI (kg/m^2^)	42.9 (31.1–53.2)	29.0 (26.2–32.5)	50.8 (44.7–56.8)	<0.001
Overweight, *n* (%)	57 (16)	57 (40)	0	
Obesity, *n* (%)	285 (77)	60 (42)	226 (100)	
Excess body weight, kg (IQR)	61 (26–92)	21 (12–31)	84 (65–105)	<0.001
*Biopsy findings*				
Fibrosis grade (IQR)	1 (0–1)	1 (0–3)	0 (0–1)	<0.001
No fibrosis*, n* (%)	156 (42)	36 (25)	120 (53)	
Grade 1*, n* (%)	122 (33)	41 (29)	81 (36)	
Grade 2*, n* (%)	32 (9)	21 (15)	11 (5)	
Grade 3*, n* (%)	22 (6)	19 (13)	3 (1)	
Grade 4/cirrhosis*, n* (%)	36 (10)	26 (18)	10 (5)	
Liver fat content, *%* (IQR)	30 (10–50)	20 (10–40)	40 (15–60)	<0.01
NAS score (IQR)^a^	4 (3–5)	4 (3–5)	4 (3–5)	<0.01
*Laboratory parameters*				
AST, IU/l (IQR)	29 (19–50)	51 (33–73)	22 (16–31)	<0.001
ALT, IU/l (IQR)	41 (25–72)	75 (50–133)	29 (19–44)	<0.001
Gamma-glutaryltransferase, IU/l (IQR)	51 (30–113)	113 (66–238)	35 (25–57)	<0.001
Bilirubin, mg/dl (IQR)^b^	0.5 (0.4–0.6)	0.5 (0.4–0.7)	0.4 (0.3–0.6)	<0.001
Albumin, mg/dl (IQR)	39 (36–41)	40 (37–43)	38 (36–40)	<0.001
Platelets, 10^9^/l (IQR)	272 (226–325)	236 (189–275)	298 (249–348)	<0.001
INR (IQR)^c^	1.0 (0.9–1.0)	1.0 (1.0–1.1)	1.0 (0.9–1.0)	<0.001

^a^Available in 336 patients (91%, missing in 32 morbidly obese patients).

^b^Available in 293 patients (80%, missing in 75 morbidly obese patients).

^c^Available in 346 patients (94%, missing in 22 morbidly obese patients).

### Factors associated with fibrosis stage

Factors associated with fibrosis stage included age and parameters suggestive of metabolic syndrome (presence of diabetes/IFG and arterial hypertension). Among laboratory parameters, AST and platelet count were associated with fibrosis stage. By contrast, INR and bilirubin were not significantly linked to fibrosis stage. We observed an inverse relationship between albumin levels and fibrosis stage, not reaching statistical significance in our total cohort (*p* = 0.056). Yet, after adjustment for patient cohort (conventional vs. morbidly obese), there was a significant inverse association between albumin and fibrosis stage. Similarly, we found a significant inverse association between BMI and fibrosis stage, which was completely abolished by adjustment for patient cohort (conventional vs. morbidly obese) and thus probably caused by different recruitment between patients with and without morbid obesity, as discussed below. The detailed analysis of underlying conditions and laboratory parameters with respect to fibrosis stages is shown in Table 2.

**Table 2:** Demographic, clinical, and laboratory parameters in relation to an ordinal model of fibrosis stage assessed by univariate analysis^a^.

Parameter	No fibrosis (*n* = 156)	Fibrosis stage				*p* value^a^	Adjusted for conventional vs. morbidly obese^b^
		I (*n* = 122)	II (*n* = 32)	III (*n* = 22)	IV (*n* = 36)		
*Demographics/underlying conditions*							
Age (years)	37 (32–55)	52 (32–59)	54 (43–63)	67 (53–73)	61 (50–66)	<0.001	<0.001
Sex (females)	95 (61%)	66 (54%)	20 (63%)	12 (55%)	17 (47%)	0.197	0.776
BMI (kg/m^2^)	46.0 (38.3–54.5)	45.1 (31.3–53.0)	31.6 (28.2–51.2)	30.9 (26.5–36.5)	33.3 (28.3–42.1)	<0.001	0.309
Diabetes/IFG*, n* (%)	63 (40)	57 (47)	18 (56)	13 (59)	28 (78)	<0.001	<0.001
Arterial hypertension*, n* (%)	87 (56)	74 (61)	15 (47)	18 (82)	32 (89)	<0.01	<0.001
Cholesterol ≥200 mg/dl*, n* (%)	55 (35)	55 (45)	17 (53)	8 (36)	8 (22)	0.77	0.346
Triglycerides ≥170 mg/dl*, n* (%)	76 (49)	72 (59)	22 (69)	15 (68)	18 (50)	0.06	<0.05
*Laboratory parameters*							
AST, IU/l (IQR)	21 (16–29)	31 (20–49)	64 (35–94)	46 (36–73)	61 (38–75)	<0.001	<0.001
ALT, IU/l (IQR)	30 (19–50)	45 (27–76)	79 (43–151)	51 (41–103)	61 (40–83)	<0.001	0.081
Albumin, mg/dl (IQR)	39 (36–41)	39 (37–42)	39 (37–43)	39 (36–43)	37 (35–40)	0.056	<0.001
Bilirubin, mg/dl (IQR)^c^	0.4 (0.3–0.6)	0.5 (0.3–0.6)	0.4 (0.4–0.6)	0.5 (0.3–0.8)	0.6 (0.5–1.0)	0.16	0.898
Platelets, 10^9^/l (IQR)	293 (243–340)	278 (239–331)	240 (200–306)	231 (204–283)	186 (100–239)	<0.001	<0.001
INR (IQR)^d^	1.0 (0.9–1.0)	1.0 (0.9–1.0)	1.0 (0.9–1.0)	1.1 (1.0–1.2)	1.1 (1.0–1.2)	0.279	0.483
*Non-invasive fibrosis assessment tools*							
NAFLD Fibrosis Score	–1.1 (–2.1 to 0.2)	–1.0 (–2.3 to 0.0)	–0.7 (–2.1 to 0.5)	–0.2 (–1.6 to 0.4)	1.3 (0.6 to 2.2)	<0.001	<0.001
FIB-4 Score	0.5 (0.4–0.8)	0.6 (0.5–0.9)	1.2 (0.7–2.0)	1.7 (1.0–2.7)	3.1 (1.7–4.7)	<0.001	<0.001
AST/ALT Ratio	0.7 (0.5–0.9)	0.6 (0.5–0.8)	0.6 (0.5–0.9)	0.8 (0.7–1.0)	1.0 (0.8–1.2)	<0.01	<0.01
APRI Score	0.15 (0.10–0.22)	0.21 (0.14–0.41)	0.60 (0.25–0.91)	0.41 (0.29–0.86)	0.77 (0.37–1.24)	<0.001	<0.001
BARD Score	2 (1–3)	1 (1–2)	1 (1–3)	2 (2–3)	3 (2–4)	<0.01	<0.001

*BMI* body mass index, *IFG* impaired fasting glucose, *INR* international normalized ratio, *NAFLD* means non-alcoholic fatty liver disease, *FIB-4* fibrosis 4 score, *AST* aspartate aminotransferase, *ALT* alanine aminotransferase, *APRI* aspartate aminotransferase to platelet ratio index.

^a^Comparisons were made by univariate ordinal regression analysis.

^b^Comparisons were made by ordinal regression analysis with group-membership (conventional vs. morbidly obese NAFLD) and the respective parameter as predictors.

^c^Available in 293 patients (80%).

^d^Available in 346 patients (94%).

### Performance of non-invasive scoring systems in patients with and without morbid obesity

**Fig. 1:**
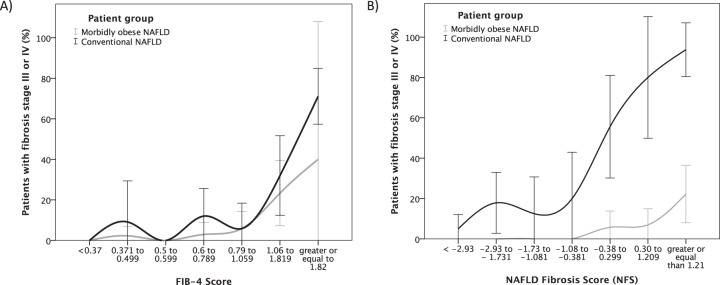
FIB-4 and NAFLD Fibrosis Score in prediction of advanced fibrosis. Association of FIB-4 and NAFLD Fibrosis Score with advanced fibrosis in NAFLD patients with and without morbid obesity.

All of the five tested scores were significantly associated with fibrosis stage in both patient cohorts (conventional vs. morbidly obese; Table 2). However, AST/ALT ratio and BARD score showed only moderate predictive potential. In our total cohort, FIB-4 score, APRI score and NFS showed the highest AUROC values in prediction of advanced fibrosis (Table 3). The AUROC for FIB-4 score (0.904) was significantly higher than AUROCs of all other scores (*p* < 0.001 for all). While FIB-4 and APRI score yielded comparable ROC curves in the conventional and the morbidly obese cohort, NFS curves differed considerably between conventional and morbidly obese NAFLD patients, thereby resulting in a lower AUROC in the total cohort. The relation of FIB-4 and NFS to the presence of fibrosis stage III/IV in our NALFD patients with respect to patient cohort (conventional vs. morbidly obese) is illustrated in Fig. 1A, B. Figure 1B shows that NFS overestimates advanced fibrosis in our morbidly obese compared to conventional NAFLD patients. The prognostic value of ROC-derived threshold values for NFS, APRI, and FIB-4 score for our total NFLD cohort is shown in Table 4.

**Table 3:** Receiver operating characteristic analyses of different non-invasive scores in prediction of advanced fibrosis (stage III/IV) in NAFLD.

Scores	Overall (*n* = 368)			Conventional NAFLD cohort (*n* = 143)			Morbidly obese NAFLD cohort (*n* = 225)		
	AUROC	95% CI	*p* value	AUROC	95% CI	*p* value	AUROC	95% CI	*p* value
NAFLD Fibrosis Score (NFS)	0.750	0.680–0.820	<0.001	0.868	0.803–0.934	<0.001	0.873	0.802–0.944	<0.001
Modified NFS (NFS_mod_)^a^	0.838	0.776–0.899	<0.001	0.869	0.803–0.934	<0.001	0.888	0.817–0.960	<0.001
FIB-4 Score	0.904	0.861–0.948	<0.001	0.871	0.805–0.937	<0.001	0.887	0.796–0.979	<0.001
APRI Score	0.848	0.801–0.895	<0.001	0.726	0.636–0.817	<0.001	0.845	0.754–0.935	<0.001
AST/ALT Ratio	0.710	0.639–0.782	<0.001	0.812	0.729–0.895	<0.001	0.698	0.586–0.810	<0.05
BARD Score	0.708	0.639–0.778	<0.001	0.833	0.758–0.908	<0.001	0.760	0.647–0.873	<0.01

*NAFLD* non-alcoholic fatty liver disease, *FIB-4* fibrosis 4 score, *AST* aspartate aminotransferase, *ALT* alanine aminotransferase, *APRI* aspartate aminotransferase to platelet ratio index.

^a^Modified NFS with BMI limited to 40 kg/m^2^.

**Table 4:** Performance of ROC-derived cut-off values for FIB-4, APRI, and NAFLD Fibrosis score in identification of advanced fibrosis in NAFLD.

Parameter	Number of patients (%)	Sensitivity (%)	Specificity (%)	Accuracy (%)	PPV (%)	NPV (%)	LRP	LRN	DOR	Youden’s J	*p* value
*Overall (n* *=* *368)*											
FIB-4 Score											
FIB-4 > 0.5	262 (71)	97	34	44	21	98	1.5	0.10	13.8	0.31	<0.001
FIB-4 > 1.0^a^	113 (31)	88	80	81	45	97	4.4	0.15	29.3	0.68	<0.001
FIB-4 > 2.0	47 (13)	57	95	89	70	92	12.6	0.45	27.9	0.52	<0.001
APRI Score											
APRI > 0.15	246 (67)	98	39	48	23	99	1.61	0.04	36.5	0.37	<0.001
APRI > 0.29^a^	128 (35)	81	72	73	35	95	2.9	0.26	11.1	0.53	<0.001
APRI > 0.9	31 (8)	32	96	86	61	88	8.46	0.70	12.1	0.28	<0.001
NAFLD Fibrosis Score (NFS)											
NFS > -3.5	337 (92)	96	9	23	17	94	1.07	0.37	2.9	0.05	0.137
NFS > –0.4^a^	159 (43)	79	64	66	29	94	2.18	0.33	6.6	0.43	<0.001
NFS > 1.8	34 (9)	24	94	83	41	87	3.74	0.81	4.6	0.18	<0.001

*FIB-4* fibrosis 4 score, *APRI* aspartate aminotransferase to platelet ratio index, *NAFLD* non-alcoholic fatty liver disease, *PPV* positive predictive value, *NPV* negative predictive value, *LRP* likelihood ratio positive, *LRN* likelihood ratio negative, *DOR* diagnostic odds ratio.

^a^Most suitable threshold according to ROC analysis and Youden’s J statistic.

### NAFLD Fibrosis Score and BMI in morbidly obese NAFLD patients

**Fig. 2:**
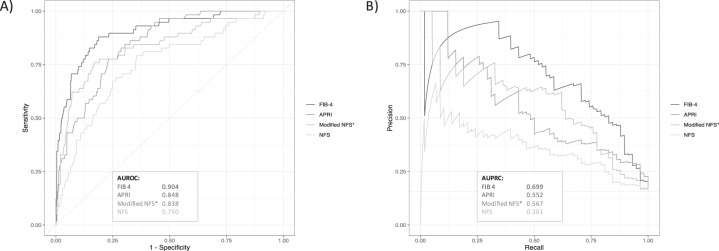
Performance of non-invasive scores in prediction of advanced fibrosis. Receiver operating characteristic and precision-recall cures for different non-invasive fibrosis scores in prediction of advanced fibrosis (stages III/IV).

Based on the aforementioned findings, we hypothesized that the excess in BMI observed in our morbidly obese cohort did not correspond to a relevant increase in the risk of fibrosis III/IV; thus, BMI may be overrepresented in the NFS when applied to morbidly obese patients. Indeed, we did observe no correlation between BMI and fibrosis stage in our morbidly obese NAFLD patients (*r* = 0.051, *p* = 0.446). Thus, we calculated a modified NFS (NFS_mod_) with no changes in the basic formula, but with BMI limited to 40 kg/m^2^. The AUROC analyses for NFS_mod_ in prediction of fibrosis stages III/IV in our conventional and morbidly obese patients are shown in Table 3. Limiting BMI to 40 kg/m^2^ led to a significant improvement of the NFS’s performance (*p* < 0.001 between AUROCs). In our total NAFLD population, the predictive value of the NFS_mod_ with regard to presence of advanced fibrosis (stage III/IV) was comparable to APRI, but still inferior to FIB-4 score (*p* < 0.001, Fig. 2).

### FIB-4 score is the most reliable predictor of advanced fibrosis

FIB-4 score improved prediction error rates as compared to all other non-invasive scores (Supplementary Fig. 1). Accordingly, FIB-4 was identified as the most potent predictor of fibrosis stage III/IV in our total cohort of NAFLD patients, independent of age, sex, BMI and group-membership (conventional vs. morbidly obese). The results of the multivariate logistic regression model are shown in Table 5. Overall, FIB-4 scores >1.0 were strongly associated with presence of advanced fibrosis (OR = 29.1 (95% CI 12.6–67.3), *p* < 0.001; Table 4). Advanced fibrosis was found in patients 45% of patients with FIB-4 values >1.0 compared to <3% when FIB-4 was ≤1.0. Moreover, probability of advanced fibrosis was 88% in patients with FIB-4 values >3.0.

**Table 5:** Multivariate logistic regression model for FIB-4 score in prediction of advanced fibrosis in NAFLD.

Parameter	Odds ratio	95% CI	*p* value
FIB-4 score	4.745	3.192–7.053	<0.001
FIB-4 score adjusted for age, sex, and BMI	3.401	2.165–5.343	<0.001
FIB-4 score adjusted for age, sex, BMI, and group-membership (conventional vs. morbidly obese)^a^	3.028	1.921–4.773	<0.001

^a^Final model: Nagelkerke *R*^2^ = 0.524, Hosmer Lemeshow Test indicated good model fit (df = 8, *p* = 0.576).

## Discussion

We were able to demonstrate in large cohort of NAFLD patients that non-invasive fibrosis scores accurately predict the presence of advanced fibrosis (stage III/IV) in both, patients with and without morbid obesity. FIB-4 score, APRI, and NFS score were identified as suitable predictors of advanced fibrosis in our NAFLD patients. Current EASL guidelines on management of NAFLD state that non-invasive scores as well as transient elastography are acceptable procedures for the identification of patients at low risk of advanced fibrosis [9]. Accordingly, in our NAFLD patients, we found high negative predictive values for ROC-derived thresholds of FIB-4 and NFS, respectively, with regard to presence of advanced fibrosis.

Assessment of fibrosis stage is of central importance in patients with NAFLD. Studies suggest that fibrosis stage is the strongest single predictor of death in NAFLD [10–12], and it has been shown that advanced fibrosis stage is associated with an increased risk of developing HCC [28, 29]. Moreover, the presence of liver cirrhosis is associated with increased perioperative risk [30, 31]. Thus, estimation of fibrosis stage should be part of the routine preoperative assessment in patients with metabolic risk profiles, and especially in morbidly obese patients. Yet, predicting liver-fibrosis in morbid obesity is challenging, as imaging techniques are often insufficient in these patients [13, 32]. Transient elastography is widely being used for non-invasive assessment of fibrosis stage; however, this strategy is hampered by high failure-rates of up to 41% in patients with morbid obesity [13], although use of the XL-probe seems to be more efficient in producing reliable results in obese NAFLD patients [33]. It has been reported that AST, male gender and presence of type 2 diabetes mellitus are associated with NASH, and waist-to-hip ratio, AST and focal necrosis on liver biopsy with fibrosis [34]. However, the applicability of non-invasive scoring systems has not been evaluated in a large cohort of morbidly obese NAFLD patients. Recently, it has been suggested that different thresholds may be required in order to identify patients with advanced fibrosis when these scores are applied to morbidly obese patients [35]. In our NAFLD patients we found that FIB-4 (and also APRI score) could be applied to morbidly obese patients without adjustments. FIB-4 score >1.0 was significantly associated with presence of advanced fibrosis in conventional (OR = 17.3 (95%CI 6.2–48.1), *p* < 0.001) and morbidly obese (OR = 32.1 (6.7–151.9), *p* < 0.001) NAFLD patients. In addition, negative predictive value was high; thus, advanced fibrosis was almost excluded, if the criterion was not met (Table 4). These findings contrast a recent study suggesting that both, FIB-4 and NFS performed poorly in morbidly obese patients [36]. In terms of identification of advanced fibrosis, AUROCs indicated good discriminative capabilities for both scores in both the conventional and the morbidly obese NAFLD patients (each group analyzed separately).

In contrast to FIB-4, NFS showed only moderate performance when applied to our total NAFLD population. BMI is a central component of the NFS; however, the NFS was derived from NAFLD patients with a mean BMI of 32.2 kg/m^2^ [15], whereas our morbidly obese group showed a median BMI of 50.8 kg/m^2^. Thus, one possible reason for the aforementioned phenomenon may be found in an overrepresentation of BMI in the NFS with consecutive overestimation of fibrosis stage in morbidly obese patients. In support of this hypothesis, we found no correlation of BMI with fibrosis stage in our morbidly obese NAFLD cohort. By contrast, BMI correlated significantly with fibrosis stage in our conventional NAFLD cohort (*p* < 0.05). Thus, we decided to calculate a modified NFS with BMI limited to a maximum value of 40 kg/m^2^ (NFS_mod_) in order to overcome the issue of fibrosis-overestimation. Indeed, this modification significantly improved the score’s performance when applied to the total cohort (Table 3). These findings support earlier reports indicating that fat distribution and metabolic syndrome rather than BMI alone are associated with NAFLD [37, 38]. Yet, our hypothesis requires further validation in other morbidly obese NAFLD cohorts.

FIB-4 score was identified as a reliable predictor of advanced fibrosis in our NAFLD patients, independent of group-membership (conventional vs. morbidly obese), age, sex, and BMI (Table 5). FIB-4 was initially developed for assessing fibrosis stage in hepatitis C/human immunodeficiency virus co-infected patients [21]. With the increasing prevalence and impact of NAFLD, this score has been increasingly evaluated with respect to prediction of advanced fibrosis in NAFLD patients [39, 40], and it has become evident that FIB-4 is indeed a valid predictor of advanced fibrosis in NAFLD. In accordance with these findings, we observed a good predictive value of FIB-4 in both our morbidly obese and conventional cohort, with comparable thresholds for predicting advanced fibrosis. Moreover, performance of FIB-4 in predicting advanced fibrosis was significantly superior to all other tested non-invasive scores. Thus, our data suggest that FIB-4 score is a useful and reliable predictor of advanced fibrosis in NAFLD patients with and without morbid obesity.

With the rise of new markers of metabolism such as adipokines/hepatokines, new parameters are available that may contribute not only to a better understanding of the pathophysiology, but also to a more accurate non-invasive assessment of NAFLD progression. In 2019, Canbay et al. proposed a score based on age, gGT, HbA1c, caspase-cleaved cytokeratin 18 fragments (M30), and adiponectin, which predicted presence or absence of NASH with a reasonable performance [41]. Moreover, adipokines and hepatokines have been suggested as potential markers for disease progression and development of HCC in NAFLD/NASH [42]. Yet, these parameters were not routinely assessed in our patients. Future studies further will have to clarify the role and potential therapeutic implications of adipokines and hepatokines in NAFLD/NASH patients.

Two main biopsy-techniques were used in our patients: needle biopsy and surgical wedge biopsy. In 2006, a study suggested that—compared to needle biopsies—wedge biopsies were associated with higher rates of fibrosis [43], which may be explained by an overestimation of fibrosis due to subcapsular sampling [44, 45]. However, wedge- and needle biopsies in the aforementioned study were not performed in the same patients. A small study comparing both techniques in the same patients showed fair to good concordance between wedge and needle biopsies with kappa coefficients ranging from 0.15 to 0.65, but also with a trend for higher fibrosis stages in the wedge samples [46]. Another smaller study—based on a morphometric evaluation—suggested that wedge biopsies were not only appropriate for assessing fibrosis stage, but also showed smaller sampling variability as compared to needle biopsy [47]. Moreover, it has been shown that even if the same biopsy-technique was used, considerable variability in the results were observed in bariatric NAFLD patients [48]. Thus, although larger comparative studies are lacking, wedge biopsy appears to be a suitable method for assessing liver fibrosis, and fibrosis assessment results seem to correlate with those obtained by needle biopsy. Yet, sampling variability remains an issue, which underlines the need for validated and reliable non-invasive tools to assess fibrosis stage in NAFLD.

This study has strengths and limitations. To our knowledge, our study represents the first comprehensive evaluation of commonly used non-invasive liver fibrosis scores in a large cohort of NAFLD patients focusing on the potential bias caused by morbid obesity. A strength of our study is that two separately recruited patient cohorts underwent histological scoring at the same central pathology. The performance of non-invasive liver fibrosis assessments in the conventional cohort was comparable to published results, indicating validity of our center’s histological evaluation. Thus, the different performance of NFS between our cohorts indeed depends on the patients’ BMI. Yet, due to the different recruitment procedures used in the two cohorts, there are significant differences in baseline characteristics that hamper direct comparability of the groups. However, the aim of this study was not to compare both patient groups in terms of their baseline characteristics, but to evaluate the performance of the non-invasive scoring systems in different stages of obesity. This is a retrospective study. However, all data were documented prospectively at the time of diagnosis/treatment in our patient data management system following the departments standard assuring high reliability of the data. Yet, bias inherent to retrospective analyses and residual confounding cannot be entirely excluded.

In conclusion, our study shows that among commonly used non-invasive scoring systems especially FIB-4 score is a useful and accurate predictor of advanced fibrosis. FIB-4 scores >1.0 are highly suggestive for advanced fibrosis in NAFLD patients of all BMI categories. NFS tends to overestimate fibrosis in morbidly obese patients. Future studies should clarify whether limitation of BMI instead of adjustment of thresholds to BMI groups may improve the score’s performance in morbidly obese patients, as suggested by our data. The use of non-invasive scoring systems may help to avoid unnecessary biopsies and identify patients requiring a closer follow-up.

## Supplementary information

Supplementary Figure Legends

Supplemental Figure

## References

[1] Sayiner M, Koenig A, Henry L, Younossi ZM (2016). Epidemiology of nonalcoholic fatty liver disease and nonalcoholic steatohepatitis in the United States and the Rest of the World. Clin Liver Dis.

[2] Sherif ZA, Saeed A, Ghavimi S, Nouraie SM, Laiyemo AO, Brim H (2016). Global epidemiology of nonalcoholic fatty liver disease and perspectives on US minority populations. Dig Dis Sci.

[3] Younossi ZM, Stepanova M, Afendy M, Fang Y, Younossi Y, Mir H (2011). Changes in the prevalence of the most common causes of chronic liver diseases in the United States from 1988 to 2008. Clin Gastroenterol Hepatol.

[4] Kabbany MN, Conjeevaram Selvakumar PK, Watt K, Lopez R, Akras Z, Zein N (2017). Prevalence of nonalcoholic steatohepatitis-associated cirrhosis in the United States: an analysis of National Health and Nutrition Examination Survey Data. Am J Gastroenterol.

[5] Williams CD, Stengel J, Asike MI, Torres DM, Shaw J, Contreras M (2011). Prevalence of nonalcoholic fatty liver disease and nonalcoholic steatohepatitis among a largely middle-aged population utilizing ultrasound and liver biopsy: a prospective study. Gastroenterology.

[6] Wong RJ, Aguilar M, Cheung R, Perumpail RB, Harrison SA, Younossi ZM (2015). Nonalcoholic steatohepatitis is the second leading etiology of liver disease among adults awaiting liver transplantation in the United States. Gastroenterology.

[7] Younossi ZM, Otgonsuren M, Henry L, Venkatesan C, Mishra A, Erario M (2015). Association of nonalcoholic fatty liver disease (NAFLD) with hepatocellular carcinoma (HCC) in the United States from 2004 to 2009. Hepatology.

[8] Torres DM, Harrison SA (2008). Diagnosis and therapy of nonalcoholic steatohepatitis. Gastroenterology.

[9] European Association for the Study of the Liver, European Association for the Study of Diabetes, European Association for the Study of Obesity. (2016). EASL-EASD-EASO Clinical Practice Guidelines for the management of non-alcoholic fatty liver disease. J Hepatol.

[10] Angulo P, Kleiner DE, Dam-Larsen S, Adams LA, Bjornsson ES, Charatcharoenwitthaya P (2015). Liver fibrosis, but no other histologic features, is associated with long-term outcomes of patients with nonalcoholic fatty liver disease. Gastroenterology.

[11] Ekstedt M, Hagstrom H, Nasr P, Fredrikson M, Stal P, Kechagias S (2015). Fibrosis stage is the strongest predictor for disease-specific mortality in NAFLD after up to 33 years of follow-up. Hepatology.

[12] Dulai PS, Singh S, Patel J, Soni M, Prokop LJ, Younossi Z (2017). Increased risk of mortality by fibrosis stage in nonalcoholic fatty liver disease: systematic review and meta-analysis. Hepatology.

[13] Castera L, Foucher J, Bernard PH, Carvalho F, Allaix D, Merrouche W (2010). Pitfalls of liver stiffness measurement: a 5-year prospective study of 13,369 examinations. Hepatology.

[14] Weiss J, Rau M, Meertens J, Hering I, Reichert L, Kudlich T (2016). Feasibility of liver stiffness measurement in morbidly obese patients undergoing bariatric surgery using XL probe. Scand J Gastroenterol.

[15] Angulo P, Hui JM, Marchesini G, Bugianesi E, George J, Farrell GC (2007). The NAFLD fibrosis score: a noninvasive system that identifies liver fibrosis in patients with NAFLD. Hepatology.

[16] Sun W, Cui H, Li N, Wei Y, Lai S, Yang Y (2016). Comparison of FIB-4 index, NAFLD fibrosis score and BARD score for prediction of advanced fibrosis in adult patients with non-alcoholic fatty liver disease: a meta-analysis study. Hepatol Res.

[17] Harrison SA, Oliver D, Arnold HL, Gogia S, Neuschwander-Tetri BA (2008). Development and validation of a simple NAFLD clinical scoring system for identifying patients without advanced disease. Gut.

[18] McPherson S, Stewart SF, Henderson E, Burt AD, Day CP (2010). Simple non-invasive fibrosis scoring systems can reliably exclude advanced fibrosis in patients with non-alcoholic fatty liver disease. Gut.

[19] Jensen MD, Ryan DH, Apovian CM, Ard JD, Comuzzie AG, Donato KA (2014). 2013 AHA/ACC/TOS guideline for the management of overweight and obesity in adults: a report of the American College of Cardiology/American Heart Association Task Force on Practice Guidelines and The Obesity Society. Circulation.

[20] Wai CT, Greenson JK, Fontana RJ, Kalbfleisch JD, Marrero JA, Conjeevaram HS (2003). A simple noninvasive index can predict both significant fibrosis and cirrhosis in patients with chronic hepatitis C. Hepatology.

[21] Sterling RK, Lissen E, Clumeck N, Sola R, Correa MC, Montaner J (2006). Development of a simple noninvasive index to predict significant fibrosis in patients with HIV/HCV coinfection. Hepatology.

[22] Denzer U, Arnoldy A, Kanzler S, Galle PR, Dienes HP, Lohse AW (2007). Prospective randomized comparison of minilaparoscopy and percutaneous liver biopsy: diagnosis of cirrhosis and complications. J Clin Gastroenterol.

[23] Wolter S, Dupree A, Coelius C, El Gammal A, Kluwe J, Sauer N (2017). Influence of liver disease on perioperative outcome after bariatric surgery in a Northern German Cohort. Obes Surg.

[24] Kleiner DE, Brunt EM, Van Natta M, Behling C, Contos MJ, Cummings OW (2005). Design and validation of a histological scoring system for nonalcoholic fatty liver disease. Hepatology.

[25] Brunt EM, Kleiner DE, Wilson LA, Belt P, Neuschwander-Tetri BA, Network NCR (2011). Nonalcoholic fatty liver disease (NAFLD) activity score and the histopathologic diagnosis in NAFLD: distinct clinicopathologic meanings. Hepatology.

[26] Glas AS, Lijmer JG, Prins MH, Bonsel GJ, Bossuyt PM (2003). The diagnostic odds ratio: a single indicator of test performance. J Clin Epidemiol.

[27] DeLong ER, DeLong DM, Clarke-Pearson DL (1988). Comparing the areas under two or more correlated receiver operating characteristic curves: a nonparametric approach. Biometrics.

[28] Hashimoto E, Yatsuji S, Tobari M, Taniai M, Torii N, Tokushige K (2009). Hepatocellular carcinoma in patients with nonalcoholic steatohepatitis. J Gastroenterol.

[29] Kawamura Y, Arase Y, Ikeda K, Seko Y, Imai N, Hosaka T (2012). Large-scale long-term follow-up study of Japanese patients with non-alcoholic Fatty liver disease for the onset of hepatocellular carcinoma. Am J Gastroenterol.

[30] Im GY, Lubezky N, Facciuto ME, Schiano TD (2014). Surgery in patients with portal hypertension: a preoperative checklist and strategies for attenuating risk. Clin Liver Dis.

[31] Nicoll A (2012). Surgical risk in patients with cirrhosis. J Gastroenterol Hepatol.

[32] Karlas T, Dietrich A, Peter V, Wittekind C, Lichtinghagen R, Garnov N (2015). Evaluation of transient elastography, acoustic radiation force impulse imaging (ARFI), and enhanced liver function (ELF) score for detection of fibrosis in morbidly obese patients. PLoS One.

[33] Myers RP, Pomier-Layrargues G, Kirsch R, Pollett A, Duarte-Rojo A, Wong D (2012). Feasibility and diagnostic performance of the FibroScan XL probe for liver stiffness measurement in overweight and obese patients. Hepatology.

[34] Ong JP, Elariny H, Collantes R, Younoszai A, Chandhoke V, Reines HD (2005). Predictors of nonalcoholic steatohepatitis and advanced fibrosis in morbidly obese patients. Obes Surg.

[35] Ooi GJ, Burton PR, Doyle L, Wentworth JM, Bhathal PS, Sikaris K (2017). Modified thresholds for fibrosis risk scores in nonalcoholic fatty liver disease are necessary in the obese. Obes Surg.

[36] Eren F, Kaya E, Yilmaz Y. Accuracy of Fibrosis-4 index and non-alcoholic fatty liver disease fibrosis scores in metabolic (dysfunction) associated fatty liver disease according to body mass index: failure in the prediction of advanced fibrosis in lean and morbidly obese individuals. Eur J Gastroenterol Hepatol. 2020. Epub ahead of print. 10.1097/MEG.0000000000001946 32976186

[37] Kwon YM, Oh SW, Hwang SS, Lee C, Kwon H, Chung GE (2012). Association of nonalcoholic fatty liver disease with components of metabolic syndrome according to body mass index in Korean adults. Am J Gastroenterol.

[38] Pagadala MR, McCullough AJ (2012). Non-alcoholic fatty liver disease and obesity: not all about body mass index. Am J Gastroenterol.

[39] Wong VW, Vergniol J, Wong GL, Foucher J, Chan HL, Le Bail B (2010). Diagnosis of fibrosis and cirrhosis using liver stiffness measurement in nonalcoholic fatty liver disease. Hepatology.

[40] Boursier J, Vergniol J, Guillet A, Hiriart JB, Lannes A, Le Bail B (2016). Diagnostic accuracy and prognostic significance of blood fibrosis tests and liver stiffness measurement by FibroScan in non-alcoholic fatty liver disease. J Hepatol.

[41] Canbay A, Kalsch J, Neumann U, Rau M, Hohenester S, Baba HA (2019). Non-invasive assessment of NAFLD as systemic disease—a machine learning perspective. PLoS One.

[42] Kucukoglu O, Sowa JP, Mazzolini GD, Syn WK, Canbay A (2021). Hepatokines and adipokines in NASH-related hepatocellular carcinoma. J Hepatol.

[43] Padoin AV, Mottin CC, Moretto M, Berleze D, Kupski C, Glock L (2006). A comparison of wedge and needle hepatic biopsy in open bariatric surgery. Obes Surg.

[44] Soloway RD, Baggenstoss AH, Schoenfield LJ, Summerskill WH (1971). Observer error and sampling variability tested in evaluation of hepatitis and cirrhosis by liver biopsy. Am J Dig Dis.

[45] Ruebner BH (1986). Collagen formation and cirrhosis. Semin Liver Dis.

[46] Rawlins SR, Mullen CM, Simon HM, Kim T, Landas SK, Walser MS (2013). Wedge and needle liver biopsies show discordant histopathology in morbidly obese patients undergoing Roux-en-Y gastric bypass surgery. Gastroenterol Rep (Oxf).

[47] Imamura H, Kawasaki S, Bandai Y, Sanjo K, Idezuki Y (1993). Comparison between wedge and needle biopsies for evaluating the degree of cirrhosis. J Hepatol.

[48] Janiec DJ, Jacobson ER, Freeth A, Spaulding L, Blaszyk H (2005). Histologic variation of grade and stage of non-alcoholic fatty liver disease in liver biopsies. Obes Surg.

