# Supplementary material for: Performance of non-invasive fibrosis scores in non-alcoholic fatty liver disease with and without morbid obesity
Source: Int J Obes (Lond). 2021 Jun 24;45(10):2197–204. doi: 10.1038/s41366-021-00881-8 (PMC8455320; doi:10.1038/s41366-021-00881-8)
Supplement: Supplementary file 1 — Supplementary Figure Legends [file 41366_2021_881_MOESM1_ESM.docx]

Supplemental Figure 1 Improvement in prediction error among different non-invasive fibrosis scores
