# Supplementary figures and images for: Performance of non-invasive fibrosis scores in non-alcoholic fatty liver disease with and without morbid obesity
Source: Int J Obes (Lond). 2021 Jun 24;45(10):2197–204. doi: 10.1038/s41366-021-00881-8 (PMC8455320; doi:10.1038/s41366-021-00881-8)

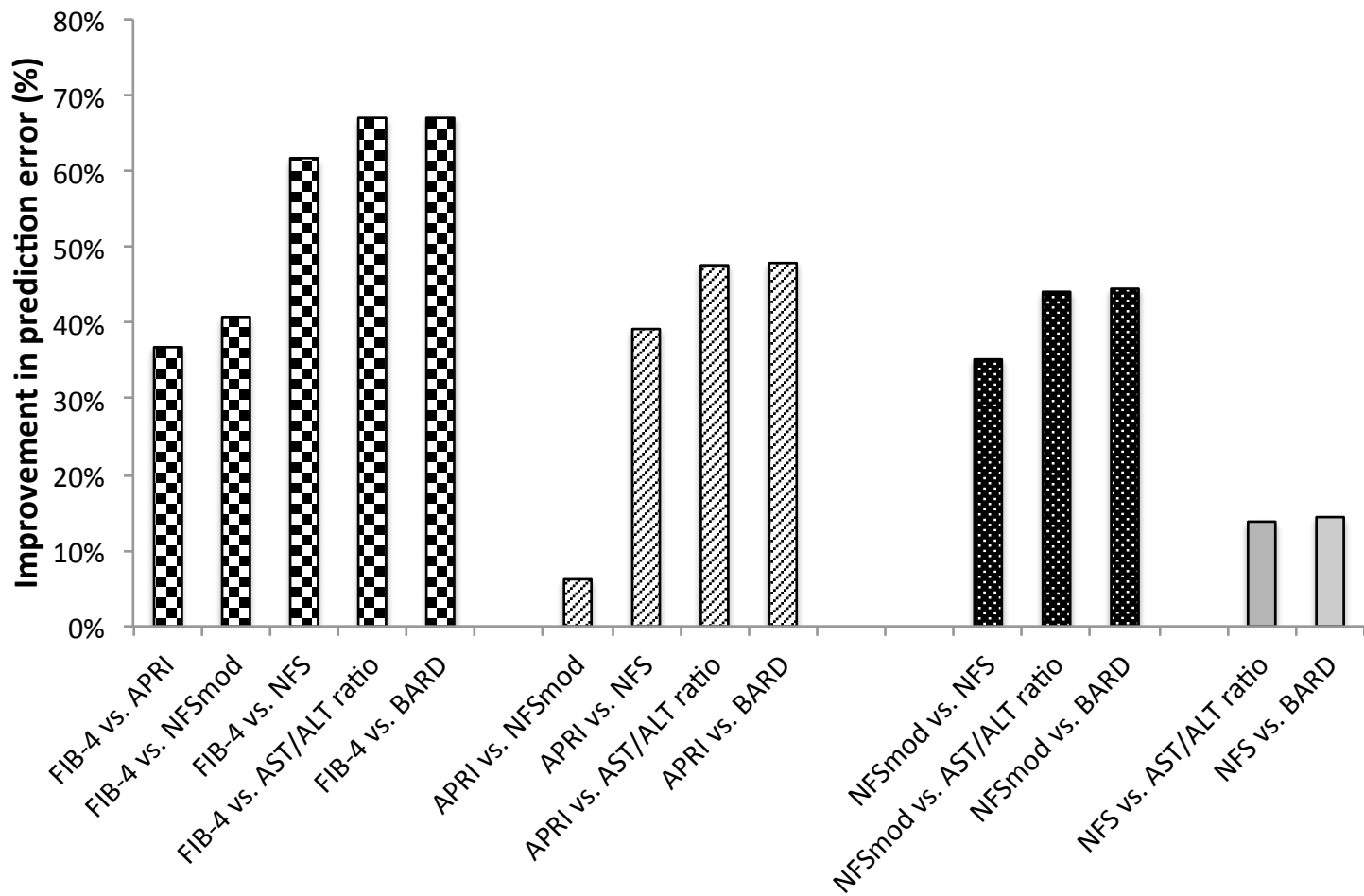

Supplement: Supplementary file 2 — Supplemental Figure [file 41366_2021_881_MOESM2_ESM.pdf]
